# Extracellularly Recorded Somatic and Neuritic Signal Shapes and Classification Algorithms for High-Density Microelectrode Array Electrophysiology

**DOI:** 10.3389/fnins.2016.00421

**Published:** 2016-09-14

**Authors:** Kosmas Deligkaris, Torsten Bullmann, Urs Frey

**Affiliations:** ^1^RIKEN Quantitative Biology Center, RIKENKobe, Japan; ^2^Graduate School of Frontier Biosciences, Osaka UniversityOsaka, Japan; ^3^Department of Biosystems Science and Engineering, ETH ZurichBasel, Switzerland

**Keywords:** microelectrode array, extracellular action potential, low-density culture, CMOS, multielectrode array

## Abstract

High-density microelectrode arrays (HDMEA) have been recently introduced to study principles of neural function at high spatial resolution. However, the exact nature of the experimentally observed extracellular action potentials (EAPs) is still incompletely understood. The soma, axon and dendrites of a neuron can all exhibit regenerative action potentials that could be sensed with HDMEA electrodes. Here, we investigate the contribution of distinct neuronal sources of activity in HDMEA recordings from low-density neuronal cultures. We recorded EAPs with HDMEAs having 11,011 electrodes and then fixed and immunostained the cultures with β3-tubulin for high-resolution fluorescence imaging. Immunofluorescence images overlaid with the activity maps showed EAPs both at neuronal somata and distal neurites. Neuritic EAPs had mostly narrow triphasic shapes, consisting of a positive, a pronounced negative peak and a second positive peak. EAPs near somata had wide monophasic or biphasic shapes with a main negative peak, and following optional positive peak. We show that about 86% of EAP recordings consist of somatic spikes, while the remaining 14% represent neuritic spikes. Furthermore, the adaptation of the waveform shape during bursts of these neuritic spikes suggested that they originate from axons, rather than from dendrites. Our study improves the understanding of HDMEA signals and can aid in the identification of the source of EAPs.

## Introduction

Action potentials (APs) are short, self-sustaining voltage transients observed in excitable membranes of several cell types. In neurons, these so-called “spikes” have been observed in the dendrite, the soma and in the axon. APs are generated at the axon initial segment (Kole et al., 2007; Meeks and Mennerick, 2007; Shu et al., 2007) and transmitted through the axonal arbor to the synapses.

APs can be non-invasively recorded by microelectrode arrays (MEAs). MEAs allow long-term recording since there is no need to break the cell membrane, as in e.g., patch-clamp, and are nowadays a common electrophysiological tool (Obien et al., 2015). After their introduction (Thomas et al., 1972; Gross et al., 1977; Pine, 1980), MEAs were used for analyzing neural network maturation (Opitz et al., 2002; Van Pelt et al., 2004; van Pelt et al., 2005), oscillatory activity in normal and pathological states (Srinivas et al., 2007), effects of electrical stimulation (le Feber et al., 2010; Weihberger et al., 2013), pharmacology (Furukawa et al., 2009), and investigating the role of specific neuronal subtypes in rhythmic oscillations (Baltz et al., 2010), among others. The availability of active, complementary metal-oxide-semiconductor (CMOS) based high-density MEAs (HDMEAs), with thousands of electrodes, allows experimentalists to record at higher spatial resolution and from multiple sites (Berdondini et al., 2009; Maccione et al., 2009; Fiscella et al., 2012; Jäckel et al., 2012; Bakkum et al., 2013; Stutzki et al., 2014).

The exact origin of the recorded extracellular action potentials (EAPs), usually identified as threshold-crossing events, is still incompletely understood (Nam and Wheeler, 2011). Therefore, we attempted to find the sources of spontaneous spiking activity in HDMEA recordings from low-density neuronal cultures. We used HDMEAs having 11,011 electrodes, with an electrode pitch of 17.8 μm, on which we plated dissociated rat neurons. In our low-density cultures, neuronal somata did not form a confluent layer on top of the electrode array, which allowed us to visually distinguish between somatic and distal neuritic sources of spiking activity. We searched for the sources of electrical activity by combining HDMEA recordings and post-hoc immunofluorescence staining for β3-tubulin, which is expressed in neuronal somata and neurites (axons and dendrites). EAPs were often recorded near somata, but also from neurites more than 100 μm away from the nearest soma. It has been shown by combination of patch clamp recordings from the soma that extracellular electrodes within 100 μm from the neuronal soma can record the cell's activity (Petersen et al., 2015). From the combined HDMEA/immunofluorescence recordings, we extracted two typical EAP shapes for somatic spikes and two for distal neuritic spikes. These typical waveshapes were used as templates for distinguishing between somatic and neuritic activity. A template-matching algorithm indicated that 14% of EAP recordings contain neuritic activity while the remaining 86% represent somatic activity. Lastly, low spike amplitude adaptation within burst events exhibited by neuritic EAPs suggests axonal, rather than dendritic identity. Our results suggest that spike shape can be used, at least partially, to distinguish between EAPs originating from the soma (axon initial segment) and (distal) axonal compartments.

## Materials and methods

### Animals

Timed pregnant Wistar rats were obtained from a commercial vendor (Nihon SLC). Animals were anesthetized with Isofluorane and sacrificed on the day of arrival to obtain embryos for primary neuron cultures. All experimental procedures on animals were carried out in accordance with the European Council Directive of 22 September 2010 (2010/63/EU) and had been approved by the local authorities (Animal Care and Use Committee of RIKEN; QAH24-01).

### Primary neuron culture

The culture protocol was adapted from Bakkum et al. (2013). The embryos (embryonal day 16–18) were extracted and euthanized by spinal cord bisection. Their brains were quickly removed and placed in ice-cold dissection medium. Cortices from two to three embryos were pooled for one plating. The cortices were isolated and enzymatically dissociated in 0.25% trypsin with ethylene-diamine-tetra-acetate (EDTA) for 20 min in a warm water bath (37°C). Cortices were washed twice for 1 min with plating medium. The tissue was mechanically triturated with a P1000 (Gilson) pipette. The cell solution was filtered through a 40 μm filter to remove aggregates and then centrifuged at 1,100 rpm for 6 min. The supernatant was removed leaving 1–2 ml of the cell pellet solution. The cells were counted and diluted to the appropriate density. 5,000 or 10,000 cells were plated on the HDMEA in a 20 μl drop centered on the electrode area. Cultures were then placed in the preservation incubator. After waiting 30 min for the cells to attach, the HDMEA chambers were slowly filled with 900 μl of plating medium. Three days later, the medium was changed for the first time by completely replacing it with growth medium. Afterwards, 30% of the growth medium was exchanged three times per week. The HDMEA chamber was covered with a membrane permeable to oxygen (O_2_) and carbon dioxide (CO_2_) but not to water vapor, bacteria or fungi (Potter and DeMarse, 2001). Cultures were kept in a humidified, preservation incubator set at 37°C and 5% CO_2_.

Dissection medium was Hank's balanced salt solution without Ca^2+^ and Mg^2+^ (Gibco, NO.14175). Plating medium was composed of Neurobasal (Gibco, NO. 21103) with 10% fetal bovine serum (Gibco, NO. 10091), 2% B27 (Gibco, NO. 17504), 1:100 Glutamax (Gibco, NO. 35050), and 1:1,000 gentamicin (Sigma-Aldrich, NO. G1397). Growth medium was Nerve Culture Medium (Sumitomo, NO. MB-X9501).

### High-density microelectrode arrays

A CMOS-based HDMEA was used for recording (Frey et al., 2010). The HDMEA has 11,011 electrodes arranged in a hexagonal pattern, with a pitch of 17.8 μm, yielding an electrode density of 3,150/mm^2^. Biocompatibility was obtained by an additional passivation of the CMOS device consisting of a stack of SiO_2_ and Si_3_N_4_ layers, as described elsewhere (Frey et al., 2010). The culture chamber was fabricated from Epoxy resin (EPO-TEK 301-2) using a PDMS stamp to protect the culture area (2.5 × 2.5 mm^2^). Three days before plating, HDMEAs were treated with oxygen plasma for 40 s at 20 W in order to render the surface hydrophilic, but also sterilize the probes. Following the plasma treatment, we took due care to keep the probes in a clean environment. The impedance of the Pt electrodes was then decreased by electrochemical deposition of Pt-black, immediately after plasma treatment, as described elsewhere, with minor modifications (Bakkum et al., 2013). The MEA surface was kept hydrophilic by filling it with sterile distilled water. The day of the culture plating, HDMEAs were sterilized by dipping the probes into 70% ethanol for half an hour. Subsequently, the HDMEAs were moved inside the clean bench, their wells were washed with sterile distilled water and finally, the probes were allowed to dry. The wells were subsequently coated with poly-D-lysine (Sigma-Aldrich, P7280, concentration of 50 μg/ml in phosphate buffered saline) for 1 h at room temperature, and washed four times with sterile distilled water afterwards. Prior to plating the cells, the electrode area was wetted with laminin (Sigma-Aldrich L2020, final concentration 20 μg/ml in phosphate buffered saline) for 20 min in the preservation incubator.

### HDMEA recordings

A total of 10 cultures were used from four different platings. The age of the cultures ranged from 14 days *in-vitro* (DIV) to 58 DIV. Previous studies have shown that both the activity of the network (Chiappalone et al., 2006) and the shape of EAPs (Weir et al., 2015) are stable after 14 DIV. Experiments were performed at least one day after a medium exchange session. HDMEAs were placed into a custom stage-top incubator with temperature, humidity, and CO_2_ control (TOKAI HIT, INU-OTOR-RE). The temperature was regulated to 36.6°C.

After transferring the plated HDMEA devices from the preservation incubator to the custom experimental incubator, the cultures were allowed to rest for half an hour, after which the recording was started. The HDMEA can simultaneously record from 126 electrodes, which were arranged in block configurations (e.g., 6 columns by 12 rows) and used sequentially to cover the whole electrode area. Each configuration's recording time ranged from 1 to 3 min using custom-made software in LabView (National Instruments).

### Immunostaining

After the recording session, the cultures were fixed for immunostaining. Cultures were washed with ice-cold phosphate buffered saline (PBS), fixed for 15 min at room temperature, followed by two PBS washes. The fixative was a freshly-prepared solution of paraformaldehyde (Sigma-Aldrich), 4% w/v in PBS. In one culture, 0.005% glutaraldehyde (WAKO Chemicals) was added. The two fixative compositions gave equal quality pictures. Immunostaining was performed as previously described (Bakkum et al., 2013), with minor modifications. Briefly, samples were permeabilized by incubation for 10 min with PBS containing 0.25% Triton-X (Sigma-Aldrich). After three washes for 5 min with PBS, samples were incubated with blocking medium for 30 min. Blocking medium consisted of 1% bovine serum albumin (BSA, Sigma-Aldrich, A4161) in PBS with 0.1% Tween-20 (Sigma-Aldrich, P1379). Primary antibodies were then added directly to the blocking solution, and the samples were left overnight at 4°C. The next day, the samples were washed three times for 5 min with PBS, and, thereafter, incubated for 1 h with secondary antibodies followed by three PBS washes for 5 min. Nuclear staining was obtained by incubation for 1 min with DAPI (300 nM in PBS), followed by three PBS washes. Samples were stored at 4°C prior to imaging. Control experiments showed that the morphology and location of soma and neurites were not affected by immunostaining.

The primary antibody used was rabbit anti-β3-tubulin (ab18207, abcam) at 1:1,000 dilution. The secondary antibody was Alexa Fluor 555 goat anti-rabbit (A-21429, Life technologies) at 1:200 dilution.

### Fluorescence microscopy and image processing

Fluorescence microscopy was performed with an Olympus BX61 epi-fluorescence microscope, equipped with a water-immersion objective (LUMPlan FL N,40x/0.8 W). Metamorph software was used for the acquisition and assembly of the images. After calibration of the Metamorph-controlled stage, successive patches were acquired and then stitched together to produce one final image for each wavelength. Filters used were RFP and infrared (IR). IR imaging allowed for visualization of the electrode surface. The images obtained at different wavelengths were aligned via Metamorph or Matlab (Mathworks) algorithms.

Further image processing was performed with Matlab and FIJI (Schindelin et al., 2012; Schneider et al., 2012). To improve the registration performance and identification of thin neurites, we processed the acquired fluorescence images in the following way. The gamma setting was modified to enhance weak intensity signals. The image was then inverted, converted to 8-bit grayscale and the levels were adjusted for the whole image. This procedure increased the intensity of the weaker signals, allowing for clearer visualization of the background electrodes and weakly stained neurites. The resolution of the final image was 0.176 μm per pixel.

The following procedure was developed to link the coordinates of the electrodes to the coordinates of the microscopy images. To accomplish this, we first registered a high-resolution image of the electrode array template to the IR image. For this image registration, affine transformation was performed by using the electrode edges at the corners of the array as control points. The RFP image was then processed with the same transformation. Before continuing with further data analysis the electrode template image was overlaid to the transformed microscopy image and observed at high magnification. We looked for instances where the electrodes on the electrode template image were misaligned to the actual electrodes of the transformed microscopy images. We evaluated the registration quality of all experiments/figures, and no such misalignment was detected.

### Generation of activity maps indicating threshold-crossing events

**Figure 1:**
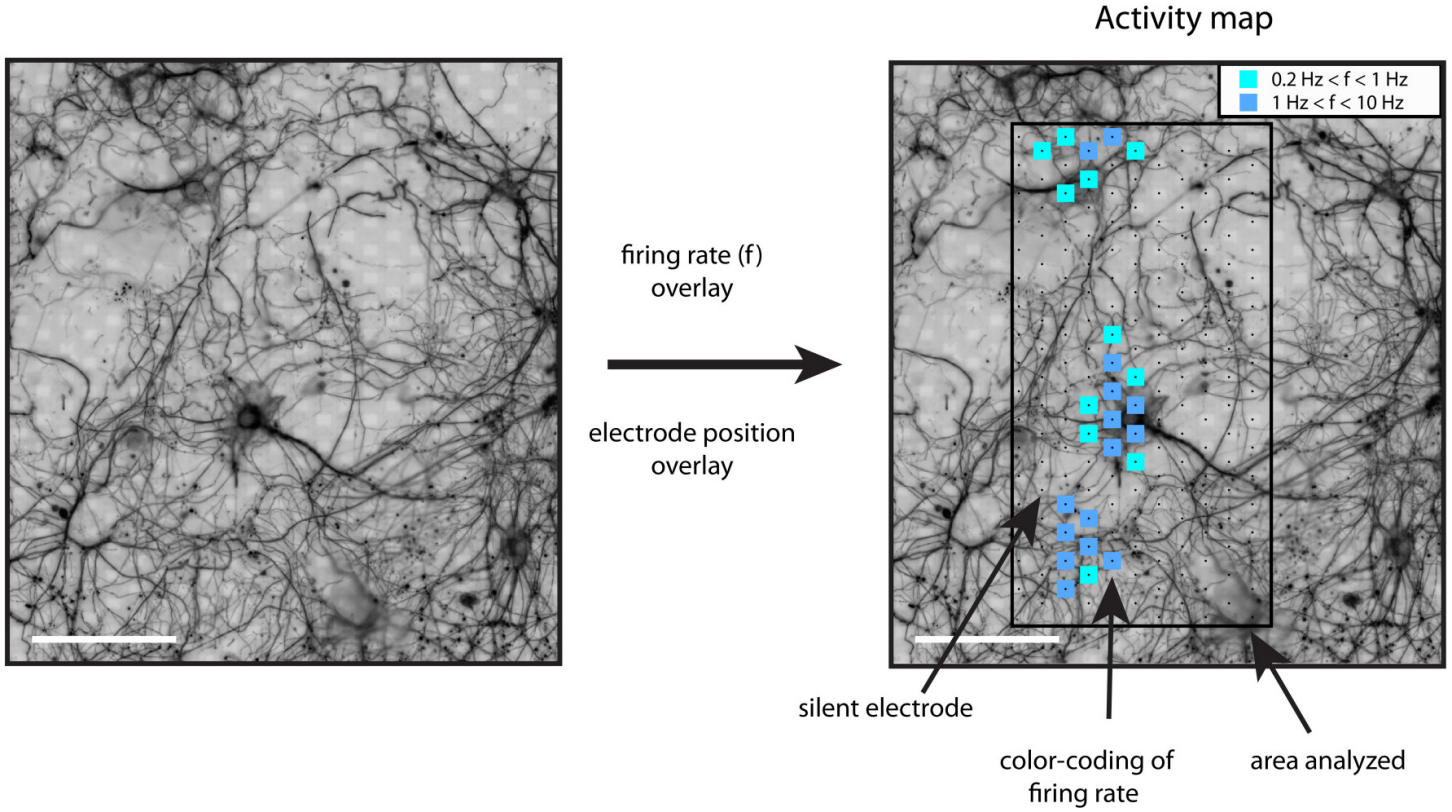
**Generation of activity maps**. The registered and processed immunostaining images were used to distinguish between somatic and neuritic sources of activity. The firing rate of each electrode was calculated with a threshold detection algorithm. The firing rate of each electrode was plotted on top of the β3-tubulin image, as color-coded squares on top of the recording electrodes. The inset shows the color code for the firing rate range, f, in Hz. Scale bars: 100 μm.

Activity maps were generated the following way. After band-pass filtering (500–3,000 Hz) of the electrode signals, events with a negative peak height larger than five times the standard deviation of the background noise were identified. Detection thresholds of five to seven times the noise levels are commonly used (Srinivas et al., 2007; Stegenga et al., 2008; Baltz et al., 2010; Sun et al., 2010). For these spiking events, the firing rate was calculated for the whole recording interval (including burst and inter-burst intervals) and for all electrodes. The resulting whole-array firing rate map was color-coded as squares centered at the electrode coordinates and overlaid on top of the immuno-fluorescence image (Figure 1). Using this procedure, areas showing spiking activity could be easily found. Further analysis was restricted to recordings showing threshold-crossing events at a rate higher than 0.2 Hz.

### Spike sorting and spike-triggered averaged footprints

Neuronal footprints, the spike-triggered averages on several electrodes, were produced based on simultaneous recording of up to 126 electrodes in a block configuration. Spike sorting was performed with three neighboring electrodes to minimize the occurrences of overlapping spike events. The three electrodes were manually selected by taking in account the activity map. Only one footprint was extracted for each block configuration.

The signals of all three electrodes were passed to UltraMegasort (UMS) (Hill et al., 2011). UMS is a freely available Matlab toolbox, which was adapted as necessary for use with HDMEA toolboxes and datasets.

UMS spike-detection was performed with a threshold-crossing algorithm, as previously described. After a threshold crossing, the algorithm was blind to other threshold crossings for 0.8 ms. The EAPs were then aligned, and 2.5 ms of recordings (50 samples per electrode at 20 kHz) of each waveform were stored. Individual spikes were aligned with respect to the negative peak of the largest spikes.

The first part of the spike sorting procedure was automatic and consisted of the following steps, already implemented by UMS: Principal component analysis (PCA) was performed, followed by K-means clustering. The interface energy similarity metric was used to identify clusters very close to each other (Fee et al., 1996). If two clusters had high interface energy, they were clustered together, and the interface energies were re-calculated until the aggregation stop criterion was reached. The aggregation stop criterion value was set to 0.001. Briefly, higher values of this criterion allow for more aggregation.

The second part of the spike sorting procedure involved the supervised evaluation of clustering. The PCA space and refractory period violations (RPV) were taken in account, so that well-isolated clusters with minimum RPV were used.

The timing of the spike-sorted activity was then used to extract signals from the neighboring electrodes of the current block configuration. A spatial footprint was generated, depicting the spike-triggered average (STA) signal for each electrode of the configuration. For this and subsequent analysis the wideband traces were used, after oversampling at 320 kHz (Blanche and Swindale, 2006). On-chip band-pass filter settings were approximately 25 Hz–3.5 kHz.

### Manual classification of footprints as somatic or neuritic

For each footprint, the electrode recording spikes with the largest negative peak was chosen as the representative electrode. The assignment of somatic or neuritic origin to a footprint was based on the distance of the representative electrode to the closest soma. If the distance was less than 50 μm, the footprint was of somatic origin, and neuritic otherwise. The identification of near-by somata was not restricted to the footprint's spatial extent or by the area of the block configuration. Instead, a region of 200 × 200 μm^2^ around the representative electrode was extracted and observed.

### Spike shape feature analysis

We compared the characteristics of somatic and neuritic spike shapes. Three spike shape features were used: the amplitude, half-width and trough-peak width. The STA signals of the representative electrode were used from each footprint. This restriction was imposed to avoid any bias resulting from duplicate data points. The amplitude of an STA was defined as the amplitude of the main negative peak. The half-width was defined as the duration of the STA main phase with values below 50% of the negative peak. The trough-peak width was defined as the time from the main negative peak to the first positive peak (Becchetti et al., 2012; Robbins et al., 2013; Weir et al., 2015). Additionally, the relative standard deviation (RSTD) was calculated as the standard deviation of the normalized traces to the peak-to-peak amplitude of the average trace (Jäckel et al., 2012).

### Statistics based on template matching

The template-matching approach represented an alternative approach of analyzing our data. Such an approach reduced any possible human bias on the classification of footprints as somatic or neuritic.

The template matching approach was used with two datasets. The first dataset consisted of the manually identified, sorted somatic and neuritic representative STA from five cultures (see Manual Classification of Footprints as Somatic or Neuritic). The second dataset consisted of 10 cultures/recordings, where spike sorting was not performed. All electrodes recording an average spike having negative peak amplitude of at least 100 μV, with an RSTD lower than 1, and more than 100 spikes were used. These constraints were imposed to reject electrodes recording spikes with low amplitudes, few spikes, or from multiple neurons.

The template matching was performed with a cross-correlation based algorithm. The cross-correlation of the spikes (from either dataset, STA or the peak-aligned mean spike respectively) to pre-extracted templates was calculated. STAs and templates were amplitude-normalized. Each spike was assigned to the template type with which it had the highest cross-correlation value.

### Spike-shape adaptation analysis

The continuous firing rate (cFR) was calculated for each spike according to the following equation:

$$\begin{array}{l} cFR\left(t\right)\;=\;d\;*\;cFR\left(t\;-\;\Delta t\right)\;+\;\left(1\;-\;d\right)\;*\;\frac{1}{\Delta t} \end{array}$$

where Δ*t* is the time between current and previous spike, and *d* is the exponential decay term (*d* = *e*^−Δ*t*/τ^), with τ = 100 ms (Stratton et al., 2012). Typically, the cFR starts at a low value and rises slowly during a burst. An exponential function was fitted to the spike shape features versus the cFR by minimizing the sum of squared residuals,

$$\begin{array}{l} g\left(x\right)\;=\;y_{c}\;*\;e^{bx}\;+\;y_{\infty } \end{array}$$

This procedure resulted in a good fit for both somatic and neuritic spikes. The adaptation rate was calculated as

$$\begin{array}{l} AR\;=\;\frac{y_{\infty }}{y_{\infty }\;+\;y_{c}}\;-\;1 \end{array}$$

and expressed in percent. The two-tailed Mann-Whitney test was used to test for differences between somatic and neuritic distributions. Reduction in spike amplitude was manifested as negative AR, while a widening of the spike was manifested as positive AR.

## Results

### Spiking activity from neuronal somata and neurites (dendrites and axons)

First, we tested the hypothesis that HDMEA electrodes can record neuronal activity from both somatic and neuritic compartments. Activity maps were generated, as described in the methods section. We noticed spiking activity, defined as threshold-crossing events, in the vicinity of and far away from neuronal somata. This suggests that the HDMEA electrodes can record EAPs originating both from somata and neurites.

**Figure 2:**
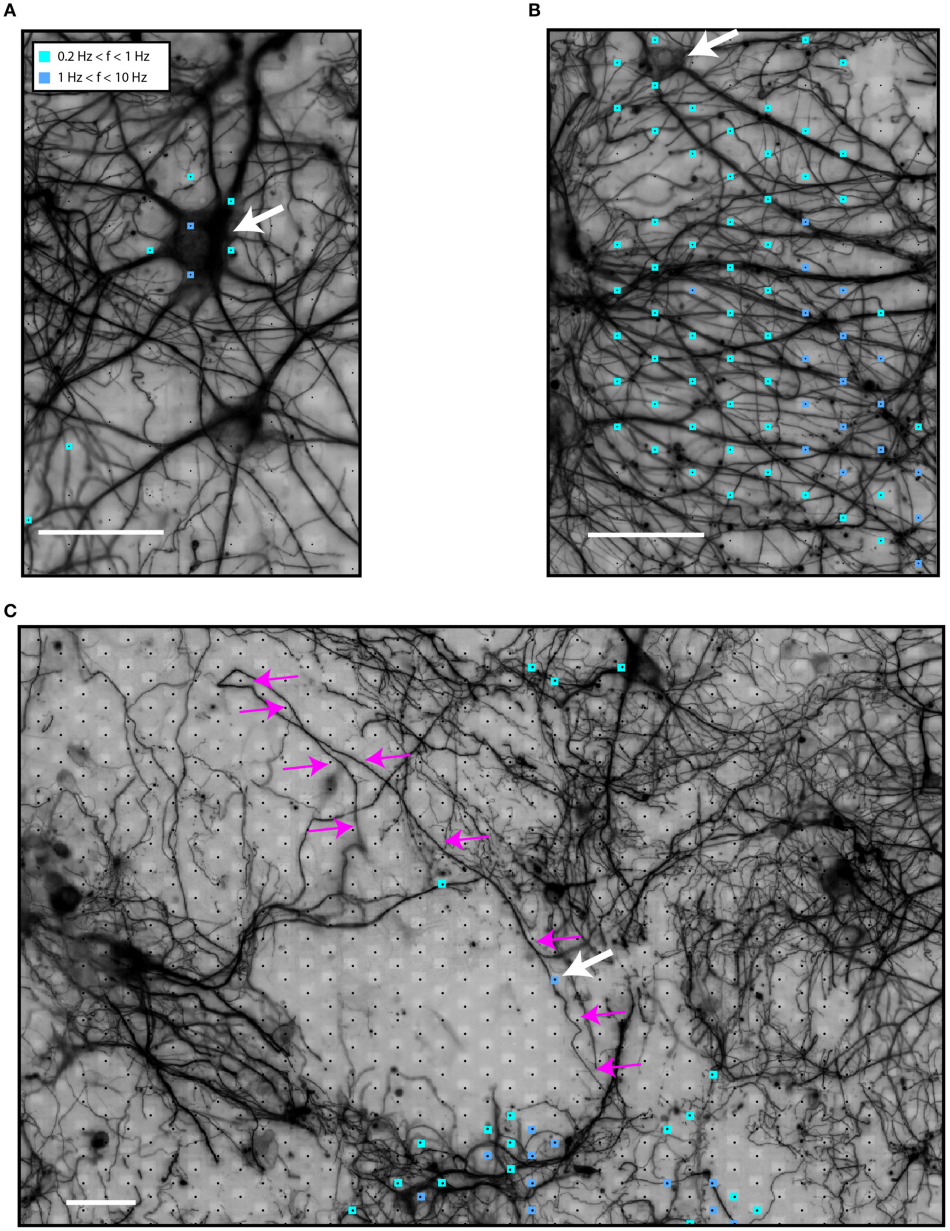
**Identification of sources of spiking activity in HDMEA recordings. (A)** An active neuron (white arrow) on top of the electrode array. **(B)** An area of high neuritic density which showed spiking activity. A soma can be seen in the upper left corner (white arrow). **(C)** A larger field of view of the electrode array with a low-density culture on top. Electric activity was recorded in areas distal to the neuronal somata. Activity from the distal part of a highlighted axon (small white arrow shows the active segment, magenta arrows highlight the axon) can be distinguished. Signals of all electrodes of the array were recorded in sequential blocks and passed through the spike-detection algorithm. Scale bars 50 μm.

Figure 2A shows electrical activity near a neuronal soma (white arrow). The variability in the firing rate of different electrodes for a putatively single neuron is because the spike-detection algorithm identified fewer spikes in electrodes further away from the spike source. As spikes are identified from threshold crossings, lower amplitudes result in lower overall spike rates. On the other hand, Figure 2B illustrates spiking events in an area with high neurite density. A soma can be seen in the upper left side (white arrow). Although no somata can be seen at the center of the area, most of the electrodes recorded spiking activity. In this case, axonal or dendritic identity could not be established due to the high neurite density. In another scenario, shown in Figure 2C, we noticed what seemed to be activity from a local, distal part of an axon (white arrow, axon traced with magenta arrows).

**Figure 3:**
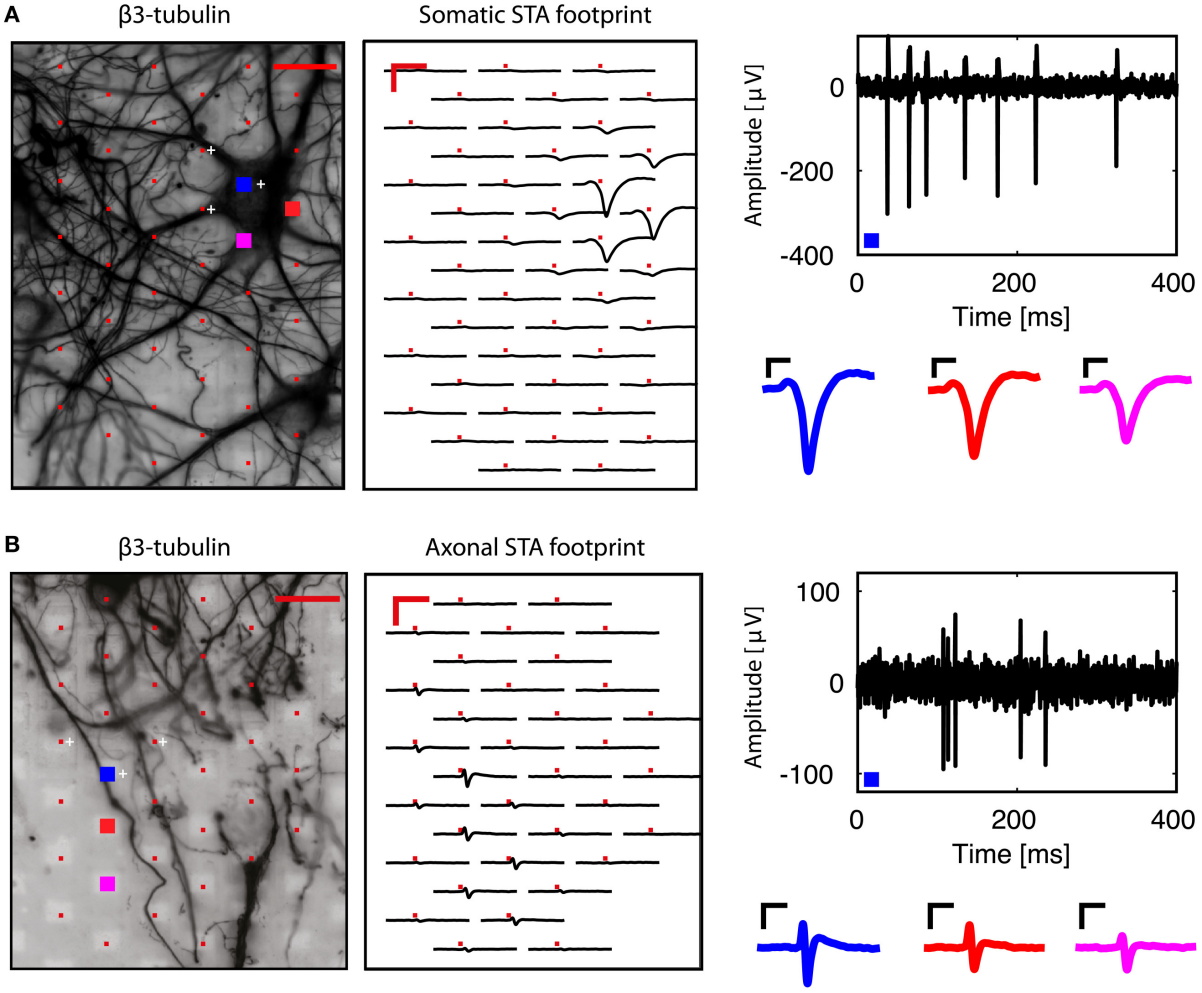
**Examples of somatic and axonal footprints**. Spike sorting and subsequent spike-triggered averaging resulted in a spatial footprint, depicting the STAs over many neighboring electrodes in the same recording block. **(A)** Examination of the signals from the neuron shown in Figure 2A. Three electrodes were used to perform spike sorting (white crosses, next to the red electrodes in the β3-tubulin image). The somatic footprint shows the STAs for the near-by electrodes, aligned on the timing of the spike-sorted activity. A band-pass (100–5,000 Hz) filtered signal from the enlarged blue electrode is shown on the right, where the negative peak can reach up to 300 μV. Three STA examples are also shown for the three enlarged (blue, red, magenta) electrodes in the β3-tubulin image. **(B)** Similar to **(A)**, but for the axonal footprint in Figure 2C. Scale bars 20 μm for β3-tubulin staining, 2 ms/200 μV for the footprints, 0.5 ms/50 μV for the STA examples.

We then analyzed each block configuration separately. We performed spike sorting and extracted single-unit activity. The footprint of each unit was obtained by averaging the wide-band, oversampled traces of 40-80 electrodes in the selected configuration. Figure 3A shows the somatic footprint from Figure 2A, while Figure 3B shows the axonal footprint from Figure 2C. For these examples, the negative peak of the somatic signals reached up to 300 μV, while that of the axonal signals reached up to 100 μV. Example STAs of three selected electrodes are also shown (enlarged blue, red, magenta electrodes in the β3-tubulin images).

We classified all footprints for which the representative electrode is 50 μm, or closer, to the nearest soma as having a ‘somatic’ and all the others as ‘neuritic’ origin. This simple procedure will likely misclassify footprints once in a while, e.g., if an axon runs below or nearby a soma, generating a detectable axonal signal. However, as we show below, in the majority of cases, this simple classification results in correct assignments. We found a total of 26 somatic and 29 neuritic footprints in five experiments from an equal number of cultures. At this stage, we did not attempt to distinguish between axonal and dendritic activity due to the high density of neurites commonly observed in the immunostaining images.

### Characterization of somatic and neuritic spike shapes

We characterized the features of somatic and neuritic spikes. From each footprint, the electrode recording the STA with the highest negative peak amplitude was selected as representative. 23 out of the 29 representative neuritic electrodes were at least 100 μm away from the nearest soma, while 6 were between 50 and 100 μm away from the nearest soma. This indicates that most of our neuritic spikes originate from distant parts of axons or dendrites. In addition, the results were not very sensitive to the limit of 50 μm, as the majority of neuritic spikes were at least 100 μm away from the nearest soma.

The same procedure was repeated for somatic footprints. In this case, the distance to a second soma was calculated. 15 out of the 26 representative somatic electrodes were between 50 and 100 μm away from a second neuronal soma, 6 were at least 100 μm away, while 5 were between 15 and 50 μm away.

**Figure 4:**
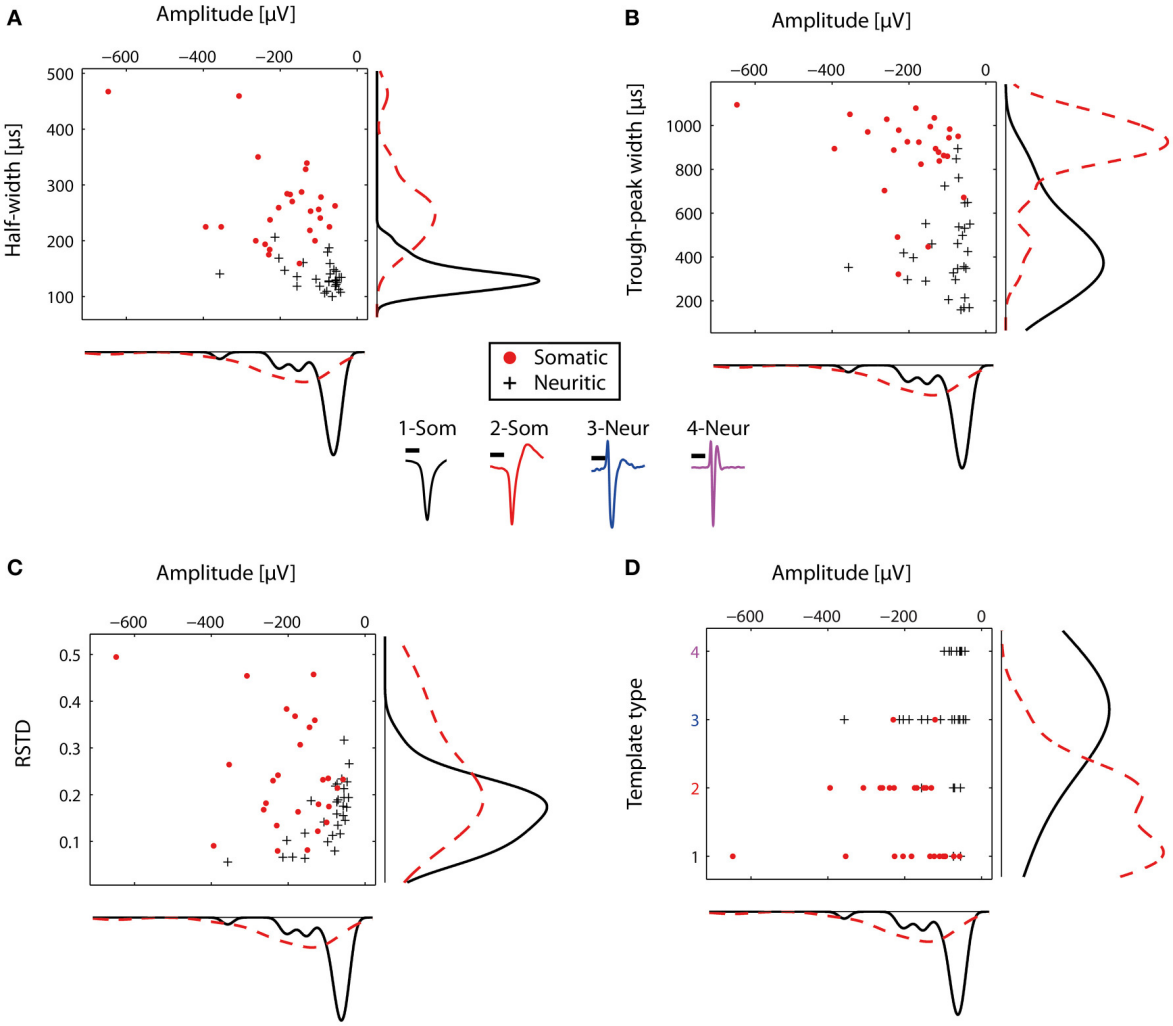
**Comparison of somatic and neuritic spike shape features**. Somata and neurites were identified by immunofluorescence. **(A)** Scatter histogram for the amplitude and half-width for the representative somatic (*n* = 26) and neuritic (*n* = 29) STAs. On the side, kernel density plots are shown. **(B)** Similar to **(A)**, but for the amplitude and trough-peak width. **(C)** Scatter histogram for the RSTD values and amplitude. Somatic STAs have wide distribution, while the neuritic STAs distribution is narrower. **(D)** Somatic and neuritic STAs were classified with a template-matching algorithm. Somatic and neuritic STAs are classified to different template types. At the center of the figure, the four template types used for classification are plotted with different colors. Each color represents a different template type, as in **(D)**. Scale bars 0.5 ms.

The STAs from the representative electrodes were then used to compare the somatic and neuritic spike shapes. The distributions of the somatic and neuritic amplitude, half-width and trough peak width were significantly different from each other (Mann-Whitney test, *p* < 0.001 for all three cases). Figures 4A,B show the distribution of the three features for each representative STA. The median of the amplitude in all somatic STAs was -171.46 μV whereas for the neuritic STAs it was -73 μV. The median of the half-width of all somatic and neuritic STAs was 250 and 130 μs respectively. The median of the trough-peak-width of the somatic and neuritic STAs was 900 and 420 μs respectively. Somatic STA amplitude, half-width, and trough-peak width distributions were not completely separated from the neuritic ones, but instead had overlapping values. Narrow somatic STAs and wide neuritic STAs were observed. Finally, the RSTD distribution of neuritic spikes was in the range of 0–0.3, non-significantly different than somatic spikes STAs (*p* = 0.0068, Figure 4C).

The typically observed somatic and neuritic STAs are shown at the center of Figure 4. STA Types 1-2 were typical of somatic spikes, while STA Types 3–4 were typical of neuritic spikes. Type 1 had a broad monophasic shape, Type 2 had a broad biphasic shape with a positive peak after the main negative peak, Type 3 was triphasic with the larger positive peak before the main negative peak. Finally, Type 4 had a narrow, symmetric triphasic shape, and it is similar to the axonal spikes reported *in-vivo* by Robbins et al. (2013).

The four described types were subsequently used as templates to blindly characterize the nature of recorded spikes in HDMEA recordings. We used a cross-correlation-based template-matching algorithm. In a first evaluation, the algorithm classified the 55 representative STAs and we compared the results against our original characterization as somatic or neuritic. 24 out of the 26 somatic STAs were classified as Type 1 or 2. 22 out of the 29 neuritic STAs were classified as Type 3 or 4 (Figure 4D). Thus, our manually identified somatic and neuritic STAs could be grouped into distinct categories by the template-matching algorithm.

**Figure 5:**
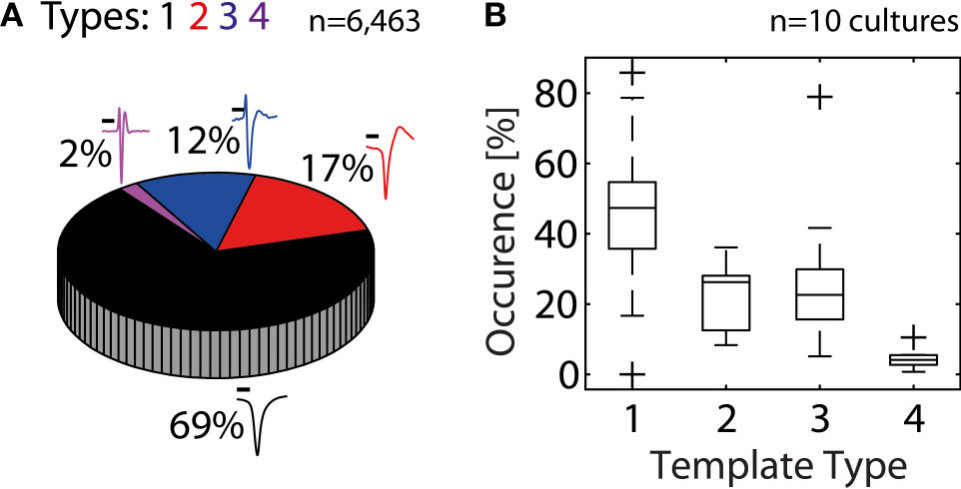
**Contribution of somatic and neuritic sources of activity in HDMEA recordings. (A)** A template-matching algorithm was used to classify recorded spikes from ten cultures/experiments as somatic (black and red templates, Types 1–2), or neuritic (Types 3–4). Data from all experiments were pooled together (*n* = 110,110). About 5.9% of all electrodes passed our constraints (6,463 electrodes). About 14% of the electrodes passing our constraints recorded neuritic activity. Scale bars 0.5 ms. **(B)** Percentage of electrodes recording different template types, per experiment (*n* = 10). For each experiment, the percentage of electrodes recording each template type was calculated. Percentage calculations were referenced to the number of electrodes passing the constraints, as shown in **(A)**. Results were grouped per template type and plotted as boxplots. Whiskers extend until 1.5 times the interquantile range. All other values are shown as outliers (+).

In the next step, we quantified the extent to which HDMEAs can record neuritic spikes. For this analysis, data from 10 cultures were used. An unsupervised approach was followed, where spikes from each electrode were identified based on threshold crossings, peak-alignment and averaging. From the total 110,110 available electrodes of all experiments, 5.9% (6,463 electrodes) passed our constraints (see Statistics Based on Template Matching). For each experiment, the number of electrodes passing our constraints ranged from 19 to 2,437 electrodes, although usual values were in the range of 200–600 electrodes. We then performed template matching of the 6,463 averaged spikes. Overall, somatic activity (Types 1–2) accounted for 86%, while the rest was assigned to neuritic (Types 3–4) activity (Figure 5A). However, the percentages of somatic and neuritic activity in the 10 individual experiments exhibited moderate variability (Figure 5B).

**Figure 6:**
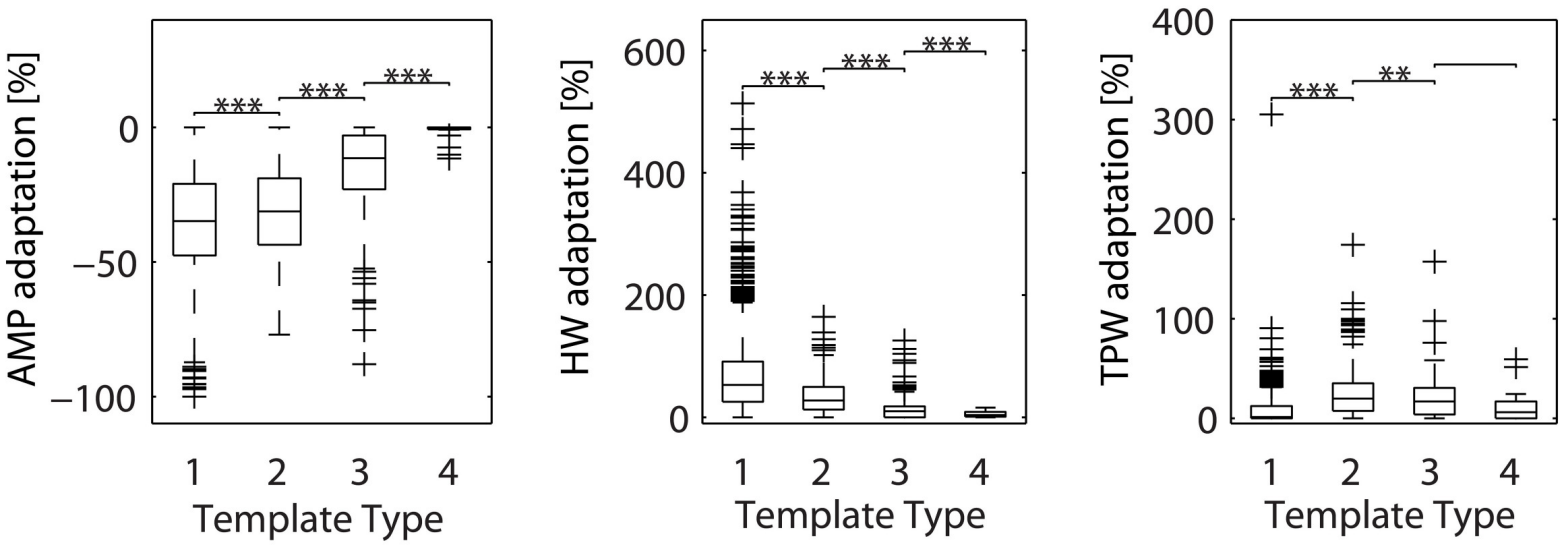
**Adaptation of spike shape features (amplitude, half-width, and trough-peak width) for the different template types**. Types 3–4, previously associated with neuritic sources, exhibit less adaptation than Types 1–2. These results were based on signals from 6,463 electrodes, from ten different cultures. For each electrode, two analysis steps were performed. Firstly, the average spike recorded by the electrode was assigned to one of the previously-defined template types with a cross-correlation based approach (see Statistics Based on Template Matching). Secondly, the adaptation rate for each feature was calculated based on individual spikes and the cFR (see Spike-Shape Adaptation Analysis). The resulting adaptation rates were pooled for all electrodes, then grouped by template type and plotted as boxplots. Whiskers extend until 1.5 times the interquantile range. All other values are shown as outliers (+). Asterisks indicate statistical significance (^**^p ≤ 0.01, ^***^p ≤ 0.001). AMP, amplitude; HW, half-width; TPW, trough-peak width.

Given our failure to separate axonal from dendritic sources with the immunostaining approach, we explored if the template matching algorithm could be used to reveal differences between the various types of templates. A possible method would be to search for differences in spike adaptation properties. Spike adaptation typically happens during bursts of spikes, where often e.g., the first spike has largest amplitude and following spikes exhibit smaller amplitudes. Previous research has shown that axonal spikes show reduced AP-shape adaptation during high-frequency firing (Meeks et al., 2005; Kole et al., 2007). On the contrary, somatic and dendritic spikes exhibit significant AP-shape adaptation (Golding and Spruston, 1998). Thus, we hypothesized that spikes of different template types would exhibit different degrees of spike-shape adaptation. We then re-analyzed the spikes from the 6,463 electrodes used in the previous analysis. For each electrode, we extracted the spike shape feature values of the individual spikes and used the cFR to produce an adaptation rate for each of the three features (see Spike-Shape Adaptation Analysis). Subsequently, the adaptation rates of each feature were grouped by template type (Figure 6). This analysis showed that somatic EAPs (Types 1–2) exhibited larger adaptation than neuritic EAPs (Types 3–4). In addition, spikes classified as Type 4, exhibited almost zero amount of adaptation supporting the hypothesis that they originate from distal axonal sources.

## Discussion

Our experiments with β3-tubulin staining and electrophysiological recordings revealed that the recorded extracellular activity could be originating from both neurites and somata. β3-tubulin stains both dendrites and axons, and a visual distinction between dendritic and axonal sources of activity could not be clearly performed due to the frequent occurrences of neuritic aggregates. We did not use dual MAP2 and Tau immunostaining because during our preliminary experiments MAP2 immunostaining had a very weak signal. On the contrary, β3-tubulin gave very good quality images. Besides the difficulties with immunostaining, we did find differences between somatic and neuritic spikes.

Our analysis shows that about 86% of the electrodes passing our constraints record somatic spikes, while 14% record neuritic spikes. When all available electrodes are taken in account, the electrodes recording somatic spikes represent 5% of the electrode population, while those recording neuritic spikes represent 0.8%. Generally, statistics of individual experiments exhibited moderate variation, while in one experiment predominantly neuritic spikes were recorded (see the Type 3 outlier in Figure 5B). This situation can occur when no neurons exist on the electrode array. This can occasionally happen because neurons seemed to attach much better in the area surrounding the electrode array, than the array itself.

Somatic spikes were typically wide, with a very small first positive peak and more pronounced second positive peak. On the other hand, neuritic spikes were narrow and had a prominent first large-amplitude positive peak. We hypothesize that most of the narrow neuritic spikes are of axonal origin. Indeed, whole-cell recordings from the axon hillock and axonal blebs have demonstrated a progressive decrease in AP half-width as the distance from the axon hillock increases (Kole et al., 2007). At approximately 10 μm from the hillock, the AP becomes narrower, reaching a half-width duration of 200 μs after 100 μm (Kole et al., 2007). On the contrary, dendritic regenerative potentials are of similar or longer duration than somatic spikes (Stuart et al., 1997). Our hypothesis is further supported by the reduced adaptation levels of neuritic spikes (Figure 6).

Our classification attempts indicate that neuritic and somatic spikes can be broadly distinguished by template-matching, albeit with some occasional misclassification (Figure 4D). The performance of template-matching depends on the chosen templates, the algorithm itself, and the spikes to be classified. Besides this, the spike adaptation levels can also be used as an indicator of the spike's axonal origin, resulting in improved classification. However, we cannot presently say that these parameters are sufficient to reliably distinguish somatic, dendritic, and axonal signals.

An interesting occasional observation is that of narrow somatic spikes (Figure 4). These spikes, although classified as somatic by our simple distance criterion, could be in reality axonal spikes, generated by axons passing below or next to a neuronal soma. The overlying silent soma increases the sealing resistance of the axonal segment, resulting in large amplitude axonal spikes, that are visually misclassified as somatic. Likewise, one possible scenario for the sporadic appearance of large-amplitude, distal neuritic spikes in our recordings would be that non-neuronal cells, possibly astrocytes, are on top of the active neurites, resulting in a similar increase in the sealing resistance (Matsumura et al., 2016).

Axonal or dendritic electrophysiology studies of dissociated cultures on HDMEAs are likely dependent on the culture conditions, such as e.g. cell density, glial growth and culture age. The presence or absence of non-neuronal cells might affect the neuritic spike amplitude recorded by HDMEA electrodes, and in turn, the percentage of somatic/neuritic spikes recorded. For example, use of cytosine arabinoside for killing proliferating cells might also reduce the probability of observing distal axonal spikes since there would be no non-neuronal cells to seal the neurites. In that case, the majority of axonal spikes are expected to be buried in noise. Recent work on detection and classification with template-matching algorithms shows that detection of even those low-amplitude spikes might be possible (Radivojevic et al., 2014; Franke et al., 2015).

Our study extends previous experimental work on the elucidation of the origin of extracellular action potentials. Claverol-Tinture and Pine recorded extracellular spikes from somata and neurites by approaching with an extracellular pipette (10–20 μm in diameter) from above the neurons (Claverol-Tinture and Pine, 2002). Consequently, the presence of a glial carpet above distal neurites prevented the routine recording from sites more than 50 μm away from the soma. On the other hand, in our microscopy analysis, all of our neuritic spikes were at least 50 μm away from the nearest soma. This may explain the difference in the reported neuritic spike half-width values, 500 μs compared to 130 μs in our experiments.

## Author contributions

KD conception, design and implementation of research; KD, TB performed experiments; KD analyzed data; KD, TB, and UF interpreted results of experiments; KD, TB, and UF wrote the manuscript.

### Conflict of interest statement

The authors declare that the research was conducted in the absence of any commercial or financial relationships that could be construed as a potential conflict of interest.
